# Whole-genome scan for selection signature associated with temperature adaptation in Iranian sheep breeds

**DOI:** 10.1371/journal.pone.0309023

**Published:** 2024-08-16

**Authors:** Zahra Patiabadi, Mohammad Razmkabir, Ali EsmailizadehKoshkoiyeh, Mohammad Hossein Moradi, Amir Rashidi, Peyman Mahmoudi

**Affiliations:** 1 Department of Animal Science, Faculty of Agriculture, University of Kurdistan, Sanandaj, Iran; 2 Department of Animal Science, Faculty of Agriculture, Shahid Bahonar University of Kerman, Kerman, Iran; 3 Department of Animal Science, Faculty of Agriculture, University of Arak, Arak, Iran; Long Island University - CW Post Campus: Long Island University, UNITED STATES OF AMERICA

## Abstract

The present study aimed to identify the selection signature associated with temperature adaptation in Iranian sheep breeds raised in cold and hot environments. The Illumina HD ovine SNP600K BeadChip genomic arrays were utilized to analyze 114 animals from eight Iranian sheep breeds, namely Ghezel, Afshari, Shall, Sanjabi, Lori-Bakhtiari, Karakul, Kermani, and Balochi. All animals were classified into two groups: cold-weather breeds and hot-weather breeds, based on the environments to which they are adapted and the regions where they have been raised for many years. The unbiased FST (Theta) and hapFLK tests were used to identify the selection signatures. The results revealed five genomic regions on chromosomes 2, 10, 11, 13, and 14 using the FST test, and three genomic regions on chromosomes 10, 14, and 15 using the hapFLK test to be under selection in cold and hot groups. Further exploration of these genomic regions revealed that most of these regions overlapped with genes previously identified to affect cold and heat stress, nervous system function, cell division and gene expression, skin growth and development, embryo and skeletal development, adaptation to hypoxia conditions, and the immune system. These regions overlapped with QTLs that had previously been identified as being associated with various important economic traits, such as body weight, skin color, and horn characteristics. The gene ontology and gene network analyses revealed significant pathways and networks that distinguished Iranian cold and hot climates sheep breeds from each other. We identified positively selected genomic regions in Iranian sheep associated with pathways related to cell division, biological processes, cellular responses to calcium ions, metal ions and inorganic substances. This study represents the initial effort to identify selective sweeps linked to temperature adaptation in Iranian indigenous sheep breeds. It may provide valuable insights into the genomic regions involved in climate adaptation in sheep.

## Introduction

Various breeds of sheep have adapted to specific regions and climates over many years in the diverse climate of Iran. Iranian sheep breeds are predominantly fat-tailed and multipurpose. However, they exhibit a range of differences, resulting in the development of 27 distinct breeds in Iran [1]. Results reported by some researchers have shown that climate [2], pathogens [3], diseases [4], diet [5], altitude [6], sunlight, temperature, UV rays, humidity, and precipitation are among the important factors that have direct effects on genome adaptation in sheep over many years. Other factors, such as digestion and the quality and quantity of forage, are indirectly influenced by weather variables (sunlight, temperature, and precipitation), and in turn, they indirectly impact the phenotypic and genetic diversity of sheep [7]. Heat stress reduces the metabolic energy of the body to the lowest possible levels by regulating the amount and type of feed consumption in sheep [5]. The morphological variation (body size and shape) in sheep is influenced by energy metabolism intake and growth in different climates [8]. Therefore, it can be concluded that weather significantly affects body weight and energy metabolism adaptation in Iranian sheep breeds. Additionally, the season or length of the day has a significant impact on reproductive efficiency, and the duration of sunlight influences physiological activity through specific mediators [9, 10].

Over the years, there have been specific changes in the patterns of variation between loci and neutral loci in the genome as a result of selection. These genetic markers in the genome, resulting from selection, are referred to as selection signatures [11, 12]. When populations are raised in different environments, natural selection may alter the frequency of a specific allele in one population, while the frequency of that allele likely remains constant in other populations. This event leads to variations in these genomic regions, which are considered indicative of positive selection in the corresponding genomic positions [13]. Today, the identification of selection signatures is one of the most intriguing areas of study for evolutionary geneticists. It offers valuable insights into the evolution of various species over extended periods [14]. Thus, it is more useful to explore the selection signature in animals selected by natural selection over many years, as well as by artificial selection in recent years, in comparison to the human selection signature [15–17].

**Fig 1:**
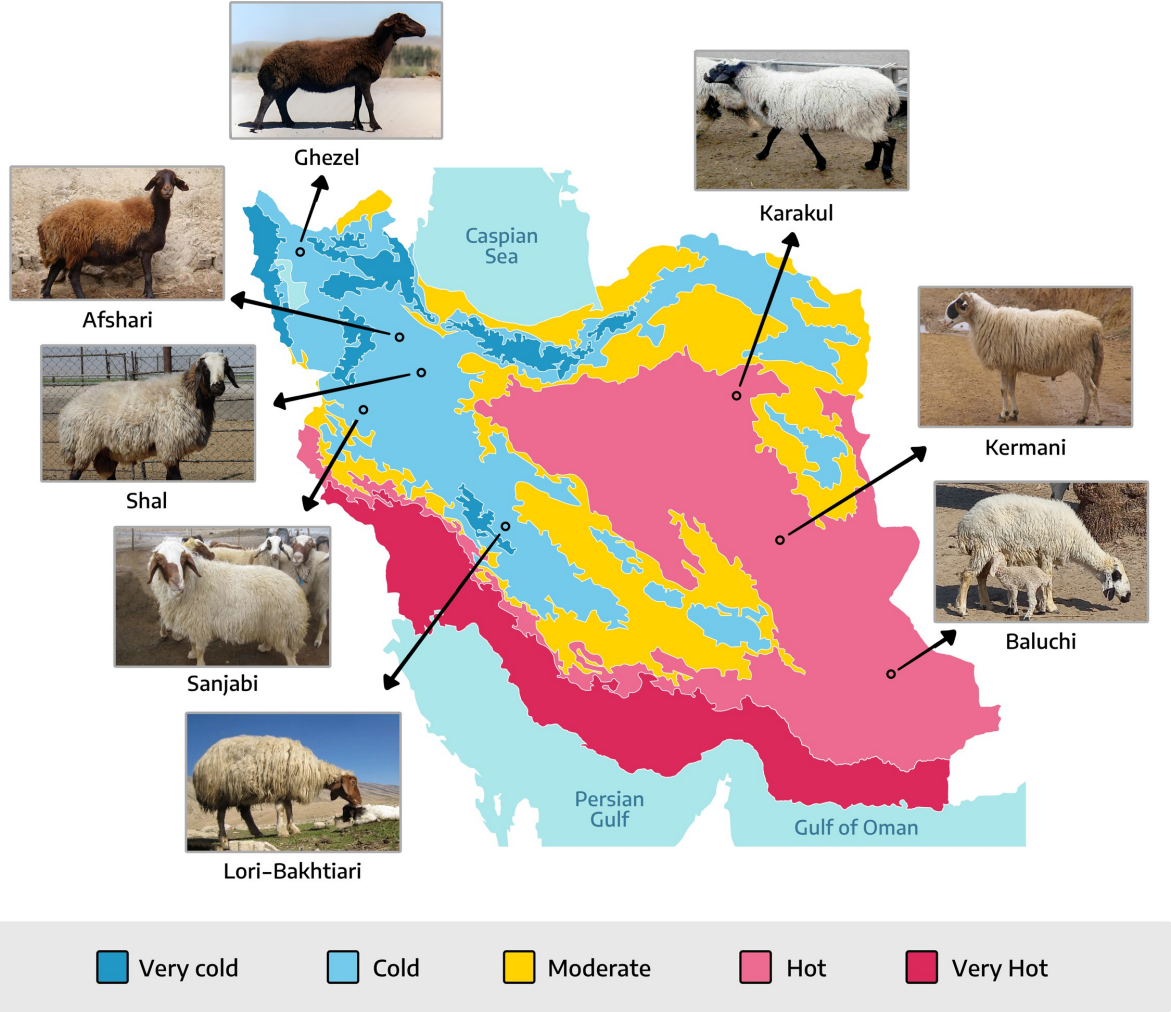
Geographical distributions of the eight Iranian sheep breeds studied.

Over the years, Iranian sheep breeds have been impacted by a combination of natural and artificial selection processes. The Ghezel, Afshari, Shall, Sanjabi, and Lori-Bakhtiari breeds are raised in the cold regions of the country, while the Karakul, Kermani, and Baluchi breeds are distributed in the warm regions (Fig 1). It can be said that Iran is one of the origins of sheep domestication, and the sheep studied in this research may be among the earliest domesticated sheep in the world. The research was conducted due to the lack of information on selection signatures associated with the adaptation of Iranian sheep to environmental conditions. Furthermore, the lack of published findings in the literature regarding the identification of genomic regions associated with environmental adaptation in sheep, especially Iranian sheep, has necessitated this study. The aim of this study was to investigate genetic markers associated with selection in specific Iranian sheep breeds, taking into account their distribution in cold and warm regions. The study also aimed to identify genomic regions that have undergone various forms of selection, whether natural or artificial, over many years.

## Materials and methods

### Ethics statement

Animal care and handling procedures were allowed and approved by the University of Kurdistan Animal Care and Use Committee (Permit No. 2020/1074). All efforts were carried out in accordance with relevant regulations to minimize any discomfort during blood collection.

### Animal samples, DNA extraction and genotyping

To identify selection signatures within the genetic makeup of native Iranian sheep breeds living in both cold and warm regions, we initially obtained temperature data for the respective regions where these animals have adapted. The information, as well as the collected samples, was obtained from the Iranian Meteorological Organization website. The examined sheep breeds were categorized into two groups based on the climate. The Ghezel, Afshari, Shall, Sanjabi, and Lari-Bakhtiari breeds were assigned to the cold group, while the Karakul, Kermani, and Balochi breeds were assigned to the hot group (Table 1). Detailed information about the Iranian sheep breeds studied, including breed names, abbreviations, phenotypic characteristics, and environmental variables, is provided in Table 1.

**Table 1:** Summary of phenotypic characteristics and environmental variables of the studied Iranian sheep.

Breed	Sampling location	Abbreviation	N (male/female)	Phenotypes and environmental variables					Agro- ecology	Average temperature (°C)	Average temperature of each group (°C)
				Coat color	Use	Horn type	Elevation (m)	Average weight (kg)			
Ghezel	Tabriz	GHE	15 (8/7)	Colored	Meat	Polled/Curved	1345	56	Cold	12.1	12.9
Afshari	Zanjan	AFS	13 (6/7)	Colored	Meat	Polled	1659.5	66	Cold	11.5	
Shal	Qazvin	SHA	15 (9/6)	Colored	Meat	Polled/Spiral	1297	70	Cold	14.2	
Sanjabi	Kermanshah	SAN	14 (7/7)	White	Meat	Polled/Rudimentary	1374	55	Cold	14.9	
Lori Bakhtiari	Shahr-e-Kurd	LRB	15 (7/8)	White	Meat	Polled/Curved	2050	70	Cold	12.2	
Karakul	Sarakhs	KAR	15 (9/6)	Colored	Skin	Polled/Spiral	281	70	Hot	17.9	20.3
Kermani	Kerman	KER	15 (9/6)	White	Wool	Polled/Rudimentary	1760	50	Hot	17.0	
Baluchi	Kahnooj	BAL	12 (6/6)	White	Wool	Polled/Rudimentary	508	45	Hot	26.0	

In each geographical area, herds consisting solely of purebred animals were identified, and a random selection of two samples (one male and one female) was obtained from each herd. Care was taken during the sampling procedure to prevent the selection of inbred animals by utilizing existing pedigree records and information provided by breeders. A total of 114 blood samples were collected from different sheep breeds, including Ghezel (n = 15), Afshari (n = 13), Shall (n = 15), Sanjabi (n = 14), Lori-Bakhtiari (n = 15), Karakul (n = 15), Kermani (n = 15), and Balochi (n = 14) (Table 1). Blood samples (2 mL per individual) were collected into the blood collection tubes containing EDTA as anticoagulant, transported to the Molecular Genetics Laboratory in the Department of Animal Science at Shahid Bahonar University of Kerman in an icebox and stored at -20°C for further analysis. The commercial DNA extraction kits (SinaPure DNA EX6001, Iran) were used to extract genomic DNA. The quality and quantity of DNA were determined using 1% agarose gel electrophoresis and a spectrophotometer (UV-1900, Shimadzu, Japan). Subsequently, the DNA samples were genotyped using the Illumina Ovine SNP 600k array, which includes 606,006 SNPs (Illumina Inc., San Diego, CA, USA).

### Data quality control

Various quality control measures implemented to ensure the accuracy and reliability of the obtained data are shown in Table 2.

**Table 2:** Description of the quality control steps.

Number of Animals	114 (61 males, 53 females)
Number of SNPs	606006
Excluding animals with more than 5% of the lost genotype	0
Excluding SNPs ≤1% MAF over all animals	31093
Excluding SNPs ≤95% call rate over all animals	16659
Excluding SNPs with unknown location	415
Remaining SNPs	557839

It should be noted that sex chromosomes were also excluded from analyses. Different stages of filtration were carried out using PLINK software version 1.9 [18]. To gain a comprehensive understanding of the population structure of the studied breeds and to identify animals that deviate from their breed group, principal component analysis (PCA) was conducted using the prcomp package in R software version 3.6.1.

### Statistical analyses

The FST statistic was used to assess the pattern of positive selection at the genome level and all loci, employing an unbiased Theta method [19] in R software version 3.6.1. The Manhattan plot was utilized to illustrate the selection signature at the genome level. Instead of considering the numerical value of each SNP, the average Theta numerical values of adjacent SNPs within a 10-SNP marker length were utilized and labeled as the Win10 value [13]. Only 0.01% of the signatures, in which all adjacent markers had high values, were identified and determined as selection signatures. Finally, the Manhattan plot was generated to identify the selection areas using Haploview software [20]. The haplotype extension FLK Single Marker method [21], also known as the hapFLK test [22], was utilized to identify selection signatures in the studied breeds. The FLK software was used to estimate the change in inbreeding coefficient and hierarchical structure under the population kinship matrix [21, 22]. The same matrix was also utilized in hapFLK, but this statistic was estimated from haplotype frequency instead of SNP allelic frequency. This method significantly enhances the ability to identify selection signatures using data obtained from high-density chips. Since the power of hapFLK is higher than that of FLK [22], this method was used for data analysis in the current study. Finally, regions of the genome that were in the 0.01 percentile of the total hapFLK values were identified as selection signatures.

### Genes and QTLs contents and gene ontology analysis

After identifying the selection signature in the genome of sheep in cold and hot regions, the genes and QTLs present in these regions were analyzed using the Biomart online databases (http://www.ensembl.org/biomart/) and the animal QTL database (https://www.animalgenome.org/cgi-bin/QTLdb/OA/index, sheep QTL database, accessed on 10 May 2022) for the OAR 3.1 genomic version of the sheep genome. To identify the genes under selection, the chromosomal position of SNPs with a high numerical value of Theta within 250 KB of the region surrounding these markers was further investigated. The function of the identified genes was investigated through an extensive search in various databases, such as Genecards (https://www.genecards.org) and OMIM (https://www.omim.org). Afterward, the DAVID online database (https://david.ncifcrf.gov) was utilized to explore the biological and functional processes of genes and to examine the ontology. Finally, the Cytoscape software version 3.5.1 was used to analyze gene networks.

## Results and discussion

**Fig 2:**
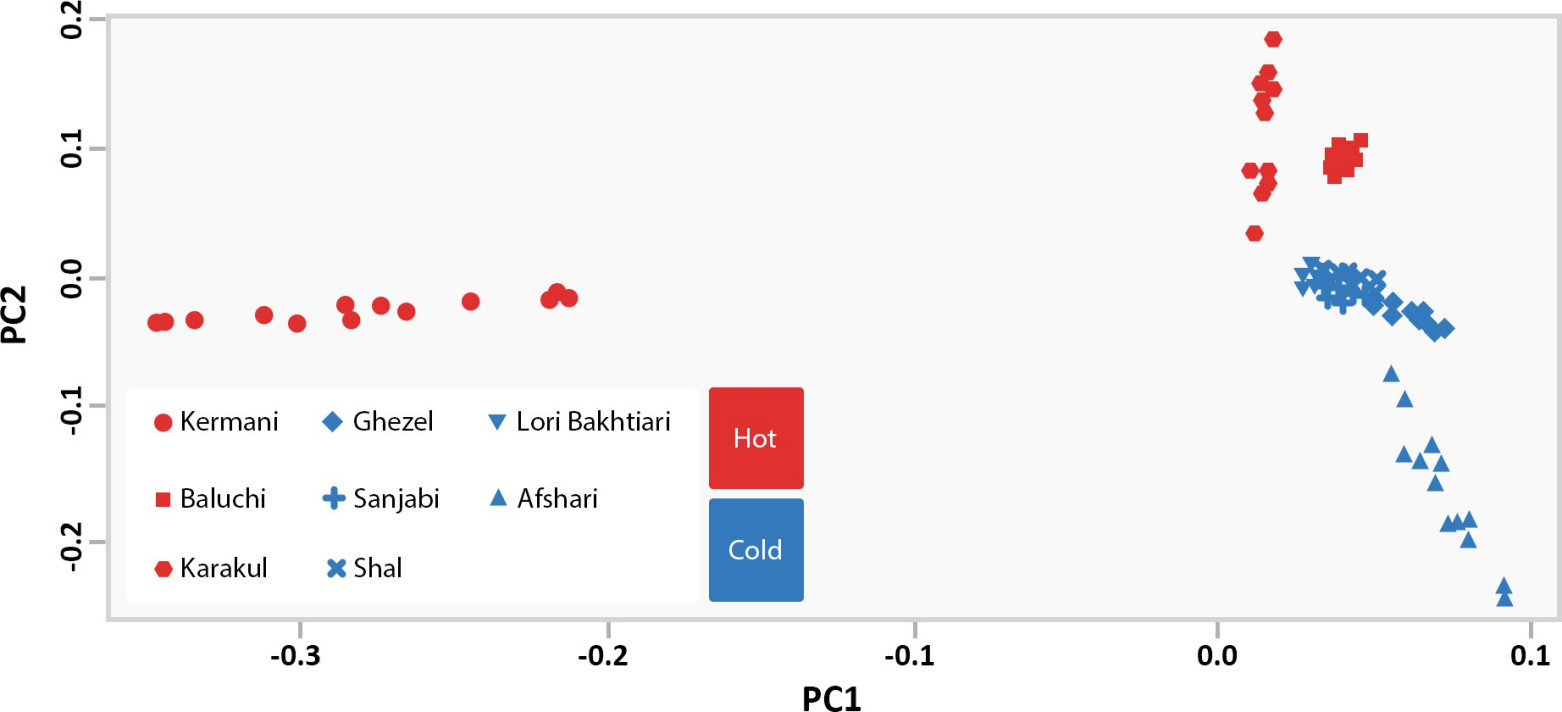
PCA plot for cold and hot climates sheep breeds. Animals from cold and hot regions are shown in blue and red, respectively.

After completing several stages of data editing, 557,839 SNP markers related to 114 animals (72 animals in cold regions and 42 animals in hot regions) were selected for further analysis. PCA analysis was conducted to examine the classification of animals into two groups based on cold and hot regions (Fig 2). In Fig 2, animals from cold regions are depicted in blue, while those from hot regions are depicted in red. The results showed that PC1 and PC2 account for 5.24% and 3.70% of the total variance, respectively. According to these two components, sheep sampled from cold and hot regions were considered as separate groups with no overlapping points.

### The selection signature detected using unbiased F_ST_ (Theta) method and bioinformatic analysis of these genomic regions

**Fig 3:**
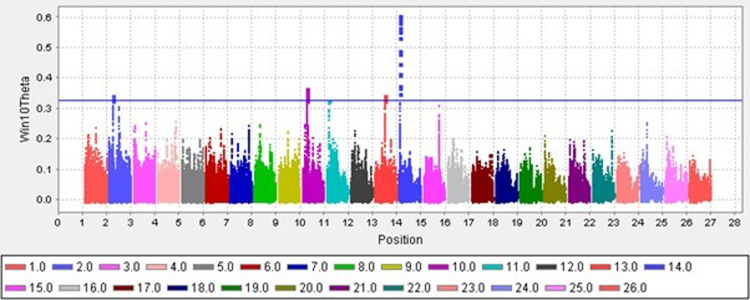
Distribution of windowed theta values for cold and hot climates sheep breeds by chromosome: SNP positions on the genome are shown on the X-axis for different chromosomes, and windowed F_ST_ (Theta) are plotted on the Y-axis.

The distribution of windowed theta values for cold and hot climates sheep breeds by chromosome is illustrated in Fig 3. As depicted, five genomic locations that surpassed the threshold and fell within the top 0.01 percentile of total Theta values were selected for further investigation. The threshold limit considered in this research was stringent to identify only the regions that exhibit the most significant population differentiation between the sheep from two groups of cold and hot regions. These regions are located on chromosomes 2, 10, 11, 13, and 14, the latter showing the strongest selection signal.

After identifying the regions under selection, the genes located in these regions were studied. The results showed that certain genes are involved in biological pathways related to the domestication and adaptation of animals to their living environment and geographical conditions (Table 3). Among the biological pathways related to the domestication of animals, the development of brain and behavioral functions, sensory perception, accumulation of pigments in tissues, immune system, and blood coagulation system can be mentioned [23]. The *TRIM62* [24], *FOXN1* [25] and *PSKR* [26] genes are located on chromosomes 11, 2, and 11, respectively, and are involved in defense and immunity responses. The *ALDOC* [27, 28], *POLDIP2* [29], and *TXNDC5* [30] genes are located on chromosomes 11, 10, and 11, respectively. They are involved in hypoxia conditions and hypoxic adaptation. The *TXNDC5* stimulates cell proliferation under hypoxic conditions, while the *ALDOC* gene participates in the stress response pathway for lung epithelial cell function during hypoxia.

**Table 3:** Genes and QTLs identified in the regions that have been under selection in cold and hot climates sheep breeds.

Chromosome- Region	Signal position	Average win10 theta	Gene name	QTL
2	233379502–233403474	0.336	*ALPG- ALP1 ‐ ALPP-ECEL1- PRSS56- CHRND- CHRNG- EIF4E2- PHC2- ZNF362- TRIM62- AZIN2*	Milk fat percentage, Meat eicosapentaenoic acid content, Hot carcass weight, Worm count, Haemonchuscontortus FEC, Change in hematocrit, Meat linolenic acid content, Body weight, Bone density, Meat docosapentaenoic acid content
10	29446648–29602556	0.34	*EEF1A1- RXFP2*	Horn type, Horn length, Horn circumference, Tail fat deposition, Somatic Cell Score, Fecal egg count, Testes weight, Fat weight in carcass, Carcass bone percentage, Carcass fat percentage, Lean meat yield percentage
11	19480094–19493823	0.326	*NLK- TMEM97- IFT20- TNFAIP1- POLDIP2- TMEM199- SEBOX- VTN- SARM1- SLC46A1- SLC13A2- FOXN1- UNC119- PIGS- ALDOC- SPAG5- PSKR-KIAA0100- SDF2- SUPT6H- SNORA70- PZGS*	Trichostrongylus adult and larva count, Internal fat amount, Milk yield persistency, Milk protein yield, Hot carcass weight, Body weight (slaughter), Milk polyunsaturated fatty acid content
13	48969775–49135367	0.335	*PPP1CC- BLOC1S5- TXNDC5*	Milk Yield, Muscle weight in carcass, Tail fat deposition
14	14207502–14242427	0.48	*SPG7- DPEP1- CHMP1A- SPATA33- CDK10 ‐ VPS9D1- FANCA- SPIRE2- TCF25- MC1R- TUBB3- GAS8*	Coat color, Fecal egg count, Dressing percentage, Bone weight in carcass, Fat weight in carcass, Nematodirus FEC

Although the aim of the current research was to identify genes related to temperature, animals located in hot and cold regions share some similarities in other characteristics such as growth rate, wool color, and physiological traits. However, they also exhibit differences. Therefore, we expected that some of the genes identified in this research may also be associated with these traits. Previous studies have shown that resistance to temperature and climate conditions is linked to other traits and genes, as mentioned below. Hence, it is essential to report these genes as well.

The results of a study by [31] showed that climate change is likely to exert strong selective pressures on traits important to body shape and organs. Additionally [32], identified candidate genes for the biological pathways of immunity, reproduction, and nervous system development to justify environmental differences. Several genes were associated with the presence or absence of horns in sheep, body temperature regulation, changes in height, and spermatogenesis. Through an examination of the signature selection in native goats and sheep in a hot and dry region [33], identified genes involved in signaling pathways and signal transmission in a wide range of cellular and biochemical processes that directly or indirectly affect adaptation characteristics. These genes are affected by dry and hot environments, such as thermal tolerance (melanogenesis), body size and growth, energy and digestive metabolism, and neural and autoimmune response. In another study [34], identified genes related to the metabolic response to stress, including the regulation of oxidative and metabolic stress and heat tolerance, to adapt to hot and dry environments. In research by [35] on environmental adaptation, genes related to immune response, morphological traits, growth and reproduction, adaptive thermogenesis, and hypoxia responses were also found in Merino sheep breeds. Furthermore [36], identified candidate genes related to meat production, immune response, and health characteristics, and discovered candidate genes for domestication and evolutionary processes for environmental adaptation.

The *SARM1*, *TUBB3*, *TMEM97*, *UNC119*, *ECEL1*, and *CHMP1A* genes are located on chromosomes 11, 14, 11, 11, 12, and 14, respectively, and are expressed in the nervous system. *SARM1* is highly expressed in the nervous system and is involved in immune response, as well as in the treatment of brain damage and neurological diseases [37, 38]. *TUBB3* plays a crucial role in the development and upkeep of the nervous system, and it is essential for guiding and maintaining axons in mammals. Furthermore, its mutation causes disorders of the nervous system and abnormalities in brain development [39]. *TMEM97* is expressed in the nervous system and in neurons that play a role in pain. It also plays a role in Alzheimer’s disease and cholesterol homeostasis [40]. Mutations in the *UNC119* gene cause defects in the nervous system [41]. *ECEL1* plays a role in regulating the respiratory nervous system and muscle motor nerves. It is expressed in the nerves and brain [42, 43]. The *CHMP1A* gene regulates the proliferation of progenitor cells in the central nervous system and plays a role in the development of the cerebellum [44].

The *CHRND*, *CHRNG* and *ALP1* genes are located on chromosome 2 and are expressed in muscle cells, where they play a role in the muscle system [45]. The ALPs play an important role in liver metabolism and skeletal growth. The *ALPG* and *ALPP* genes are located on chromosome 2 and expressed in the placenta. They are involved in miscarriage, placental, and infant weight loss [46, 47]. The *SEBOX* gene, located on chromosome 11, plays a crucial role in embryonic cell division, egg maturation, early embryogenesis, and pre-implantation growth [48].

The *TCF25* and *MC1R* genes, located on chromosome 14, play a major role in regulating the coat color of animals. The melanocortin 1 receptor (*MC1R*) influences a range of skin and hair pigmentation. This gene plays a crucial role in regulating the synthesis of eumelanin (black-brown) and phaeomelanin (red-yellow) in mammalian melanocytes. Active mutation leads to an increase in eumelanin synthesis [49]. The *TCF25* gene is located near *MC1R*, which has been implicated in the coat color of yellowish-brown sheep [50].

The *TMEM199* and *SLC13A2* genes are located on chromosome 11 and are involved in liver function. Deficiency of *TMEM199* leads to liver diseases, and mutations in it result in fatty liver [51, 52]. *SLC13A2* plays a role in glucose and energy metabolism in mammals and is a potential target for treating obesity, fatty liver disease, and type 2 diabetes. It is also expressed in the kidney and urinary system and is the primary factor influencing urinary citrate excretion [53, 54]. The *DPEP1* gene and *CHMP1A* gene are located on chromosome 14 and regulate kidney diseases [55]. The *SLC46A1* gene is located on chromosome 11 and is responsive to dietary folate restriction [56].

*PIGS* (Entrez Gene), *ZNF362* [57], and *FANCA* [58] genes are located on chromosomes 11, 2 and 14, respectively, and play a role in blood diseases.

*FOXN1* plays a crucial role in organogenesis, thymus development, regulation of keratin gene expression, and skin growth and development. Its mutation causes a bald phenotype in mice and humans [59, 60].

The *AZIN2* gene is located on chromosome 2 and is involved in heart growth and development. It is also expressed in the brain and testes, playing a role in regulating testosterone levels and sperm motility [61, 62]. *PPP1CC* is located on chromosome 13 and plays a crucial role in spermatogenesis. Reduction or absence of *PPP1CC* can lead to male infertility [63].

*PRSS56* and *UNC119* genes are located on chromosomes 2 and 11, respectively. *PRSS56* plays a role in eye development [64], while *UNC119*, which is found in the retina, is involved in the release mechanism of the photoreceptor neuron. It has also been identified in other parts of the body, including the liver, kidneys, brain, and fibroblasts. Additionally, it has been found to play a crucial role in the function of the T cell receptor (*TCR*) [65, 66].

Some genes, such as *POLDIP2* [67], *IFT2* [68], *NLK* [69], *SPG7* [70], *SNORA70* [71], *VTN* [72], *TNFAIP1* [73], *TXNPC5* [74], and *PHC2* [57], are involved in cell division and cell physiology. The *RXFP2* gene, located on chromosome 10, is involved in the development of the horned phenotype in sheep [75].

The *TRIM62* [76], *KIAA0100* [77], *TXNPC5* [78], *DPEP1* [79], *TNFAIP1* [80], *CHMP1A* [81], *AZIN2* [82], *GAS8* [83], *CDK10* [84], *FANCA* [58], *EEF1A1* [85], *ELF4E2* [86], and *SPAG5* [87] are involved in tumor development and various cancers.

In addition to the identified genes, QTLs were also identified in the regions under selection (Table 3). These QTLs were mostly associated with traits related to the acidity of meat, changes in hematocrit, body weight, bone density, horn characteristics, fat deposition in the tail, carcass characteristics, milk production and composition, traits related to color and coverage percentage, and the number of fecal eggs. The presence of these QTLs and the associated genes in the cold and warm categories indicates the variations and diversity in traits within the two groups being studied.

To gain a better understanding of the genes under selection, gene ontology analysis was also conducted (see Table 4). The results showed that genes are involved in biological pathways, the establishment of organelle localization, single-organism processes, the establishment of spindle localization, cell division, and cellular response to calcium ions. The pathways identified in this study are directly or indirectly associated with adapting to environmental conditions. For example, the calcium ion, as a second messenger, plays an important role in the stimulus-response reactions of cells. This process activates cellular reactions by maintaining low cytoplasmic Ca2+ concentration under resting conditions and mobilizing Ca2+ in response to stimuli. The role of Ca2+ as a second messenger was first discovered in the stimulation and contraction of skeletal muscle. The characteristics of Ca2+ as a messenger include a variety of target molecules, rapid mobility capability, a tendency to establish localization, and the ability to create a generalized cellular response. This ion plays a role in the physiological contractions of skeletal, cardiac, and smooth muscles [88].

**Table 4:** Analysis of gene ontology and enriched pathway terms in regions under positive selection.

GO Term	Biological process	p-Value	Genes
GO:0051640	Organelle localization	0.024	*CHMP1A-IFT20-SPAG5-SPIRG2*
GO:0044699	Single-organism process	0.049	*POLDIP2-FANCA-SEBOX-SHCBPI-SUPT6H-TNFAIP1-AZIN2-CHMP1A-CHRND-CDK10-DEF8-DPEP1-ECE1-FOXN1-GAS8-IFT20-NLK-PIGS-PHC2-PRSS56-SLC13A2-SPAG5-SPIRE2-UNC119-VTN*
GO:0051293	Establishment of spindle localization	0.058	*SPAG5-SPIRE2*
GO:0071277	Cellular response to calcium ion	0.071	*CPNE7-DPEP1*
GO:0051301	Cell division	0.084	*CHMP1A-SPAG5-SPIRE2*
GO:0051656	Establishment of organelle localization	0.095	*CHMP1A-SPAG5-SPIRE2*

### Identification of selection signature using the hapFLK method and bioinformatic analysis of these genomic regions

**Fig 4:**
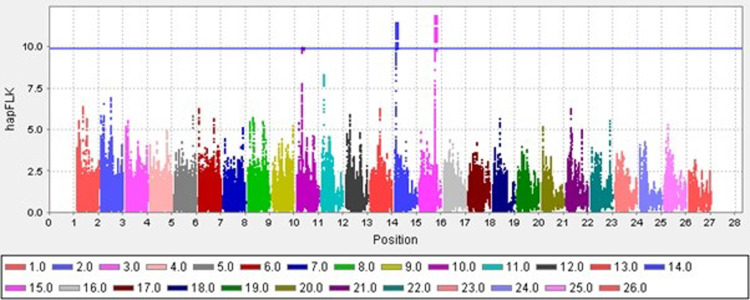
Distribution of windowed hapFLK values for cold and hot climates sheep breeds by chromosome: SNP positions in the genome (bp) are shown on the X-axis for different chromosomes, and windowed hapFLK are plotted on the Y-axis.

Three genomic regions that were in the top 0.01 percentile of all hapFLK values were identified and determined to be selection signatures (Fig 4). These regions are located on chromosomes 10, 14, and 15, with the highest selection signal being on chromosome 15.

After identifying the regions under selection, the genes within these regions were also examined. The results of the present study showed that many genes identified by the applied methods are common. This study identifies genomic regions that may be associated with environmental adaptation in Iranian sheep. Previous research suggests that genomic regions identified by multiple statistical methods are more likely to be valid candidates for the traits under study [13, 89]. Regarding the function of genes commonly identified by these two statistical methods in this study (Table 5), explanations are provided in the previous section. Genes not commonly identified include *FRY*, a transcription activator located on chromosome 10 (Entrez Gene). The *ANKRD11* gene on chromosome 10 is a chromatin regulator that is essential for neural growth. It plays a fundamental role in the growth and function of the skull, face, teeth, skeleton, and central nerves. Its mutations are associated with intellectual disability and skeletal abnormalities [90, 91]. *SHCBP1* gene [92] on chromosome 14, the *EXT2* [93] and *CD82* [94] on chromosome 15 play a role in suppressing cancer.

**Table 5:** The identified genes and QTLs published under selection in cold and hot climates sheep breeds.

Chromosome	Signal position	Average hapFLK	Gene name	QTL
10	29436435–29446648	9.92	*RXFP2-FRY*	Muscle weight in carcass, Lean meat yield percentage, Carcass fat percentage, Muscle weight in carcass
14	14133106–14204663	10.85	*SHCBP1- SPATA33- ZNF276- GAS8- SPATA2L- DEF8- SPG7- CPNE7- DPEP1- SPIRE2- CENPBD1- CDK10- CHMP1A*	Coat color, Fecal egg count, Dressing percentage, Bone weight in carcass, Fat weight in carcass, Total lambs born, Nematodirus FEC
15	72530500–72698511	11.12	*EXT2-CD82*	Hind leg length, Staple length

The results of QTL analysis indicated that the QTLs were mostly associated with carcass characteristics, color and percentage of body cover, number of fecal eggs, total lambs born, and leg characteristics. The presence of these QTLs and the associated genes in the cold and hot climate groups indicates the variations and diversity in traits within the two groups being studied.

To gain a better understanding of the functions of genes under selection, a gene ontology analysis was performed (Table 6). The results revealed that genes are implicated in biological process pathways, including cellular response to calcium ions, cell division, organelle fission, cellular response to metal ions, and cellular response to inorganic substances. Some pathways were identical to those in the Theta results. The explanation of the calcium ion was provided earlier. Studies have demonstrated that inorganic substances play a crucial role in cellular immunity [95]. Metal ions such as Ni^2+^, Co^2+^, Cu^2+^, and Cr^3+^ play a significant role in the immune system, and excessive amounts of these ions may lead to adverse immune responses (autoimmunity) and cellular reactions [96, 97]. Exposure to certain metals may lead to carcinogenesis and oxidative stress in cellular responses [98].

**Table 6:** Analysis of gene ontology and enriched pathway terms in regions under positive selection.

GO Term	Biological process	p-Value	Genes
GO:0071277	Cellular response to calcium ion	0.0387	*CPNE7-DPEP1*
GO:0000280	Nuclear division	0.0555	*FANCA-CHMP1A-SPIRE2*
GO:0048285	Organelle fission	0.0632	*FANCA-CHMP1A-SPIRE2*
GO:0071248	Cellular response to metal ion	0.0864	*CPNE7-DPEP1*
GO:0071241	Cellular response to inorganic substance	0.0954	*CPNE7-DPEP1*

To some extent, the findings of this study have been corroborated by other research. In a study [99], stated that during climate change, animal organisms alter their gene expression and metabolism to elevate the concentration of various anti-stress compounds. Moreover, they alter their physiology, growth, and reproduction in response to climate change, leading to rapid adaptation and evolution at the population level. In a study conducted by [100], the results also indicated that climate change likely imposes significant selective pressures on key traits, body appearance, and organs.

Therefore, it can be stated that the results of the present study can provide valuable information for future research to identify differentiating genes, pinpoint candidate genomic regions for important economic traits, and gain a better understanding of the biological mechanisms involved in the evolutionary adaptation of Iranian sheep breeds to climatic conditions. However, further investigations are necessary to identify the functions of genes and related QTLs. In general, this study had some limitations, including sample size and racial diversity, which means that further research in independent samples is needed to confirm the identified genomics conclusively. Furthermore, further investigations are required to identify the functions of genes and associated QTLs.

## Conclusion

In this study, a genomic investigation was conducted to identify the selection markers in sheep from the cold and hot regions of Iran. Examining the genes in the selected regions revealed their association with traits related to responses to cold stress, heat, the nervous system, cell metabolism, the immune system, cell division, gene regulation and expression, skin and fetal development, skeletal growth, adaptability to hypoxic conditions, horn development, cancer, etc. The identified genes are likely associated with biological pathways related to animal domestication, including the development of brain and behavioral functions, pigmentation, adaptation to living environments and geographical conditions, and milk production. Furthermore, the identified QTLs were mainly associated with growth traits, body weight, bone density, horn characteristics, carcass and tail traits, milk traits, color-related traits, and coverage percentage, among others. These two groups have adapted to different environmental and geographical conditions and also differ functionally, making their differences seem logical. Some genes do not have a well-established biological role, and there may be potential mutual effects that are not yet understood in this context. Therefore, to determine the precise role of these genes, it is essential to conduct further comprehensive functional studies and biological system analyses.
